# Paired clinical 12 lead and apple watch electrocardiogram data repository from childhood cancer survivors authors

**DOI:** 10.1016/j.dib.2026.112452

**Published:** 2026-01-08

**Authors:** Oguz Akbilgic, Ibrahim Karabayir, Luke Patterson, Stephanie B. Dixon, Daniel A. Mulrooney, Kirsten K. Ness, Melissa M. Hudson

**Affiliations:** a Department of Cardiovascular Medicine, Wake Forest School of Medicine, Winson-Salem, NC, USA; b Department of Oncology, St Jude Children’s Research Hospital, Memphis, TN, USA; c Epidemiology and Cancer Control, St Jude Children’s Research Hospital, Memphis, TN, USA

**Keywords:** Electrocardiogram, Apple watch, Clinical ECG, Wearables, smartwatch

## Abstract

Childhood cancer survivors (CCS), exposed to prior cardiotoxic treatments such as anthracyclines and chest radiation, are at lifelong risk of cardiovascular complications. Current guidelines recommend periodic echocardiographic surveillance, but adherence rates are as low as 41%. This dataset provides paired same-day 12-lead clinical electrocardiograms (ECG) and single-lead wearable ECG recordings from the Apple Watch, collected from adult CCS participating in the St. Jude Lifetime Cohort Study (SJLIFE). The availability of paired wearable and clinical ECGs enables the development and validation of remote AI-based cardiac screening tools, potentially leading to more precise long-term cardiovascular surveillance in this population. Using this dataset, researchers can assess whether an AI model developed using clinical ECG can be repeat when using ECG from an Apple Watch.

Specifications Table

**Table:** 

Subject	Health Sciences, Medical Sciences & Pharmacology
Specific subject area	Electrocardiography, Wearable Devices, Childhood Cancer Survivorship
Type of data	ECG waveform files (NumPy format), demographic spreadsheet (.xlsx)
Data collection	Clinical ECG: GE MUSE X system; Apple Watch ECG: Apple Watch Series 7 using custom app “ECG-Air”
Data source location	St. Jude Children’s Research Hospital, Memphis, TN
Data accessibility	Repository name: SJLIFE-Paired-Clinical-and-Apple-Watch-ECG-Repository Data identification number: 10.5281/zenodo.16536261 Direct URL to data: https://github.com/akbilgic/SJLIFE-Paired-Clinical-and-Apple-Watch-ECG-Repository Instructions for accessing these data: through the provided link where data is publicly available
Related research article	Butler L, Ivanov A, Celik T, Karabayir I, Chinthala L, Hudson MM, Ness KK, Mulrooney DA, Dixon SB, Tootooni MS, Doerr AJ, Jaeger BC, Davis RL, McManus DD, Herrington D, Akbilgic O. Feasibility of remote monitoring for fatal coronary heart disease using Apple Watch ECGs. Cardiovasc Digit Health J. 2024 Apr 5;5[3]:115–121. doi: 10.1016/j.cvdhj.2024.03.007. PMID: 38,989,042; PMCID: PMC11232422.

## 1 Value of the Data

• Enables validation of AI-based ECG models across clinical and wearable modalities.
• Supports development of remote cardiac screening tools for childhood cancer survivors.
• Facilitates research on domain adaptation and signal quality comparison between ECG sources.
• To best of our knowledge, there is no other such dataset available in publicly available nor research specific domains
• This data has been used to test clinical and wearable ECGf or only one AI model. However, this data set can be used to test concordance of any other AI model, providing a unique research opportunity.

## 2 Background

Childhood cancer survivors (CCS) face lifelong cardiovascular risks due to prior exposure to cardiotoxic treatments like anthracyclines and chest radiation [1]. These therapies, while life-saving, can cause progressive cardiac damage [2]. Although guidelines recommend routine echocardiographic surveillance every 2 to 5 years, adherence remains low—often due to barriers such as limited access to care, cost, and low health literacy. This highlights the need for scalable, low-cost, and remote screening alternatives [3].

Recent advances in artificial intelligence (AI) applied to electrocardiography (ECG-AI) have shown promise in detecting cardiac dysfunction using single-lead ECGs [[4], [5], [6], [7], [8]]. Simultaneously, wearable devices like the Apple Watch now enable real-time, at-home ECG monitoring. However, a major challenge is validating whether AI models trained on high-fidelity 12-lead ECGs perform reliably on single-lead wearable data.

To address this, we present a novel dataset of same-day paired 12-lead clinical ECGs and Apple Watch ECGs from adult CCS enrolled in the St. Jude Lifetime Cohort Study (SJLIFE). This resource supports comparative studies between clinical and wearable ECGs, enabling future research in AI model development, validation, and domain adaptation for remote cardiovascular screening.

## 3 Data Description

Participants in this dataset were drawn from the St. Jude Lifetime Cohort Study (SJLIFE), an ongoing longitudinal study (NCI U01CA195547, MPI Hudson & Ness) designed to investigate long-term health outcomes in survivors of childhood cancer. SJLIFE is conducted by St. Jude Children’s Research Hospital and includes 5-year cancer survivors diagnosed and treated for a pediatric malignancy at St. Jude. The cohort is rigorously characterized through comprehensive clinical assessments conducted during in-person follow-up visits. These assessments span multiple domains including cardiovascular, metabolic, neurocognitive, and psychosocial health [9].

The dataset includes paired electrocardiographic recordings from 243 adult survivors of childhood cancer, each consisting of a standard 12-lead clinical ECG and a single-lead Apple Watch ECG. All recordings were collected during the same clinical visit, as part of the SJLIFE Cohort. The dataset is structured to facilitate side-by-side comparison of clinical and wearable ECGs from the same individuals, captured within a short time interval (File Name: shared_paired_data_243.csv).

### 3.1 ECG acquisition protocol

Paired electrocardiographic data were collected in a controlled, sequential protocol. Each participant underwent two ECG recordings during the same clinical visit including a standard 12 lead clinical ECG and a single lead smartwatch ECG. Raw 10-second digital 12-lead electrocardiograms were obtained from using the GE MUSE system’s XML export module. SJLIFE ECGs were recorded at either 500 Hz or 250 Hz and were down sampled to 250 Hz by linear interpolation to create uniform inputs. Single lead smartwatch ECG was collected using an Apple Watch which was uniformly sampled at 512 Hz. More details about both clinical and smartwatch ECGs are provided below.

### 3.2 Clinical 12-lead ECG (File name: clinicalecgs_full_243)

• Conducted using the GE MUSE X system.
• Performed in the supine position with standard electrode placement.
• Data were exported in XML format for subsequent processing.
• The waveform data were parsed out from XML and saved into a NumPy file to be shared in GitHub.

### 3.3 Apple watch ECG (File name: appleecgs_full_243)

• Immediately following the clinical ECG, electrodes were removed, and participants were asked to sit upright.
• Participants were fitted with a study-provided Apple Watch Series 7 on their left wrist.
• They were instructed to record a 30-second ECG using the built-in ECG application.
• The data were retrieved in JSON format using a custom mobile application (``ECG-Air'') developed by the study team for secure data export.
• The waveform data were parsed out from JSON and saved into a NumPy file to be shared in GitHub.

The mean time interval between clinical ECG and Apple Watch ECG recordings was 42 ± 18 min. This standardized approach ensures that the paired ECGs—though derived from different postures and devices—were recorded within a clinically relevant time window, minimizing the likelihood of physiologic variation due to temporal factors.

## 4 Experimental Design, Materials and Methods

The dataset presented in this manuscript was collected as part of an existing ancillary study (NCI 1R01CA261834, MPI Akbilgic & Hudson) consists of paired clinical 12-lead (MAC1200, GE Healthcare, Milwaukee, WI) and Apple Watch (Cupertino, CA) single-lead ECGs were recorded from participants during their SJLIFE clinical evaluation since December 2022. The paired clinical and smartwatch ECG datasets were previously used to test for correlation of two ECG modalities within an ECG-AI model for fatal coronary heart disease prediction [8]. A total of 243 participants from SJLIFE.

## Limitations

Not Applicable.

## Ethics Statement

A consent form was collected from participants for smartwatch ECG data collection and sharing. The study was approved by St Jude Children’s Hospital Ethical Review Board (IRB00084229).

## CRediT Author Statement

**OA, IK, KKN:** and MMH designed the study. OA created the first draft. IK and OA prepared the GitHub data repository. All authors reviewed the manuscript and provided feedback.

## Data Availability

GitHubSJLIFE-Paired-Clinical-and-Apple-Watch-ECG-Repository (Original data).

## References

[1] Dixon S.B., Liu Q., Chow E.J. (2023). Specific causes of excess late mortality and association with modifiable risk factors among survivors of childhood cancer: a report from the Childhood Cancer Survivor Study cohort. Lancet.

[2] Ehrhardt M.J., Liu Q., Mulrooney D.A. (2024). Improved cardiomyopathy risk prediction using global longitudinal strain and N-terminal-pro-B-type natriuretic peptide in survivors of childhood cancer exposed to cardiotoxic therapy. J. Clin. Oncol..

[3] Yan A.P., Chen Y., Henderson T.O. (2020). Adherence to surveillance for second malignant neoplasms and cardiac dysfunction in childhood cancer survivors: a childhood cancer survivor study. J. Clin. Oncol..

[4] Mahajan R., Kamaleswaran R., Howe J.A., Akbilgic O. (2017). Cardiac rhythm classification from a short single lead ECG recording via random forest. 2017 Comput. Cardiol. (CinC): IEEE.

[5] Karabayir I., Davis R., Chinthala L. (2024). Single lead wearable ecg simulation augmented with ai for heart failure identification. J. Am. Coll. Cardiol..

[6] Akbilgic O., Butler L., Soliman E.Z., Chang A.C., Limon A. (2024). Intelligence-Based Cardiology and Cardiac Surgery.

[7] Karabayir I., Wilkie G., Celik T. (2024). Development and validation of an electrocardiographic artificial intelligence model for detection of peripartum cardiomyopathy. Am. J. Obstet. Gynecol. MFM.

[8] Butler L., Ivanov A., Celik T. (2024). Feasibility of remote monitoring for fatal coronary heart disease using Apple Watch ECGs. Cardiovasc. Digit. Health J..

[9] Howell C.R., Bjornard K.L., Ness K.K. (2020). Cohort Profile: the St. Jude Lifetime Cohort Study (SJLIFE) for paediatric cancer survivors. Int. J. Epidemiol..

